# An Unusual Case of Macrophage Activation Syndrome (MAS)- Hemophagocytic Lymphohistiocytosis (HLH) Triggered by Necrotizing Autoimmune Myopathy

**DOI:** 10.7759/cureus.38501

**Published:** 2023-05-03

**Authors:** Tyiesha S Brown, Gregory Vo, Prangthip Charoenpong

**Affiliations:** 1 Internal Medicine, Louisiana State University Health Sciences Center, Shreveport, USA; 2 Critical Care Medicine, Louisiana State University Health Sciences Center, Shreveport, USA; 3 Pulmonology and Critical Care, Louisiana State University Health Sciences Center, Shreveport, USA

**Keywords:** medical critical care, critical care, s: dermatomyositis, acute polymyositis, hemophagocytic lymphohistiocytosis (hlh), macrophage activation syndrome (mas)

## Abstract

Macrophage activation syndrome (MAS)- hemophagocytic lymphohistiocytosis (HLH) secondary to inflammatory myopathies such as dermatomyositis (DM), polymyositis (PM), and necrotizing autoimmune myopathy is exceedingly rare in the medical literature. We present the complicated diagnosis and treatment of a 41-year-old female who presented with proximal muscle weakness and shock. Following an extensive critical care workup, she was diagnosed with MAS-HLH, triggered by a newly diagnosed necrotizing autoimmune myopathy. In this case report and literature review, we would like to highlight the importance of recognizing the clinical signs of MAS-HLH in rheumatological disorders and the necessity for rapid treatment.

## Introduction

Hemophagocytic lymphohistiocytosis (HLH) is a rapidly progressive inflammatory storm that has a high mortality if untreated. Currently, the major causes of secondary HLH include malignancy and infection. Rheumatological disorders make up a small subset of HLH cases and are generally secondary to systemic lupus erythematosus (SLE) and adult-onset Still disease [1]. Macrophage activation syndrome (MAS)-HLH secondary to inflammatory myopathies is exceedingly rare in the medical literature. We present the complicated diagnosis and treatment of a MAS-HLH patient following a new diagnosis of necrotizing autoimmune myopathy.

## Case presentation

A 41-year-old woman with a past medical history of diabetes and hypertension presented with fever, periorbital maculopapular rash, diffuse myalgias, arthralgias, and chills for three weeks. Subsequently, she developed proximal muscle weakness in her upper and lower extremities, causing difficulty completing her activities of daily living. The patient was found to have leukocytosis and a fever. Despite empiric antibiotics, she remained febrile and developed hypotension, requiring vasopressors. She was transferred to our facility for further evaluation.

**Figure 1:**
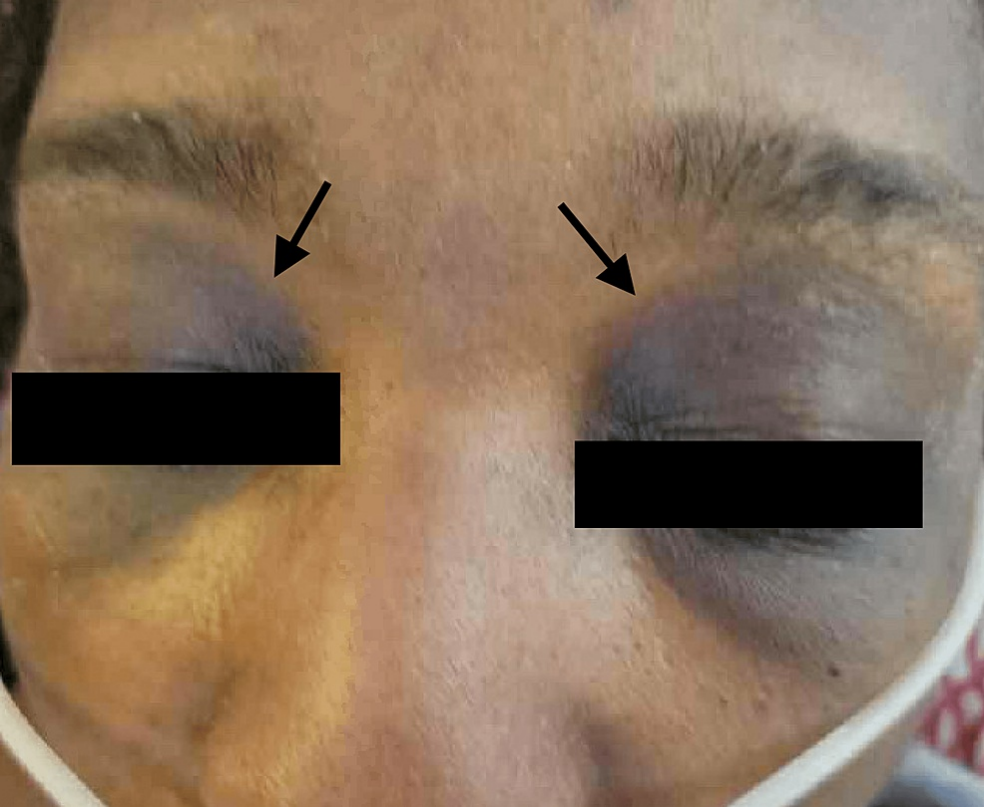
Symmetric periocular hyperpigmented macular skin changes.

**Figure 2:**
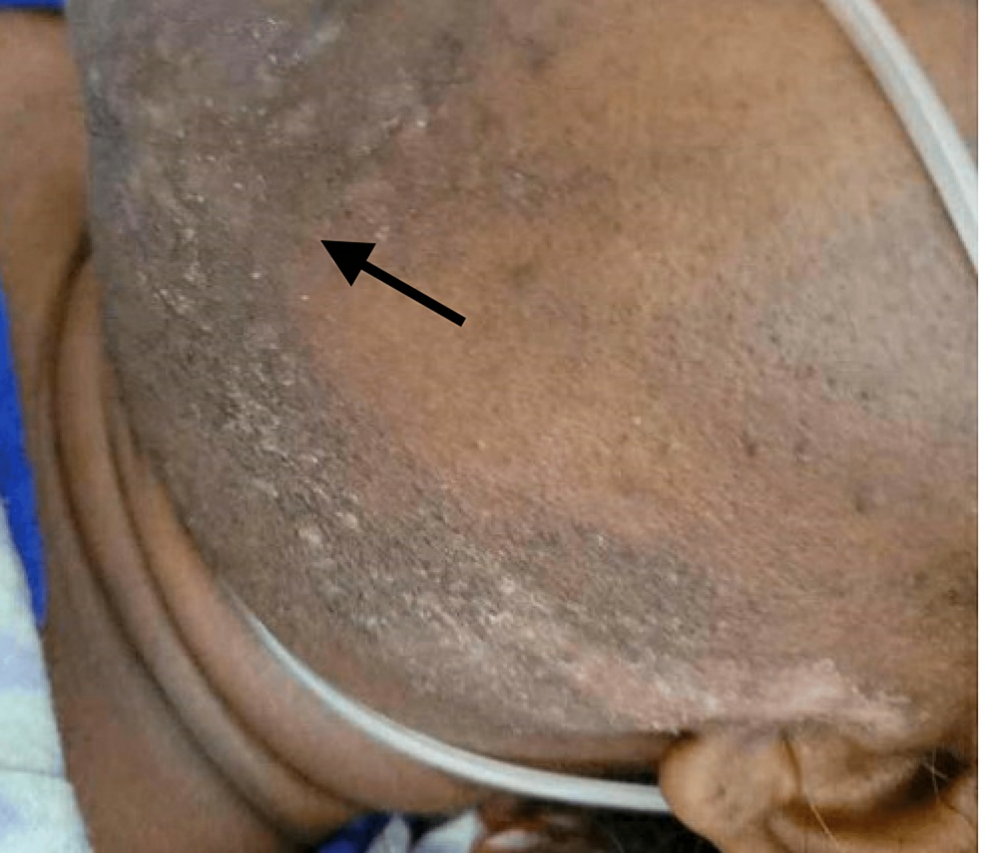
Asymmetric lichenoid skin changes with hyperpigmentation and hypopigmentation.

Physical examination was notable for bilateral subconjunctival hemorrhages, bilateral axillary lymphadenopathy, and a maculopapular rash affecting the periocular areas (Figure 1), face (Figure 2), thorax, and lower extremities. Upper and lower extremity proximal muscle weakness was also noted. Laboratory findings included leukocytosis, anemia, acute kidney injury, mild elevation of liver function test, and elevated erythrocyte sedimentation rate (ESR), C-reactive protein (CRP), D-dimer, and procalcitonin. Empiric broad-spectrum antibiotics and antifungal coverage were initiated, and a comprehensive infectious workup was unremarkable. As infection was unlikely, rheumatologic and hematologic disorders were of concern.

A complete rheumatological panel for autoantibodies, muscle biomarkers, flow cytometry, and excisional axillary lymph node, skin, and bone marrow biopsies was obtained. A rheumatologic workup revealed elevated serum aldolase, creatine phosphokinase (CPK), lactate dehydrogenase (LDH), and a positive anti-Jo-1 antibody (Table 1).

**Table 1:** Laboratory values during hospitalization *Lab not collected on admission **Lab not recollected WBC: white blood cell; AST: aspartate aminotransferase; ALT: alanine transaminase; CRP: C-reactive protein; CPK: creatine phosphokinase, LDH: lactate dehydrogenase

Lab Finding	On Admission	A Few Days Later	Prior to Discharge
WBC ( K/uL)	17.78	.26	5.20
Hemoglobin ( g/dL)	8.7	6.9	8.8
Platelets ( K/uL)	247	18	204
Ferritin ( ng/mL)	>40,000	>40,000	4,707
Creatinine ( mg/dL)	1.50	2.70	.75
AST ( U/L)	61	1,805	15
ALT ( U/L)	25	408	33
CRP ( mg/dL)	18.60	26.20	2.58
LDH ( U/L)	691	>4,000	288
CPK ( U/L)	185	939	48
Fibrinogen (mg/dL)	360	80	454
Triglycerides ( mg/dL)	*	439	**
CD25 (soluble IL-2 receptor) ( pg/mL)	*	7609.9	2,158.8

**Figure 3:**
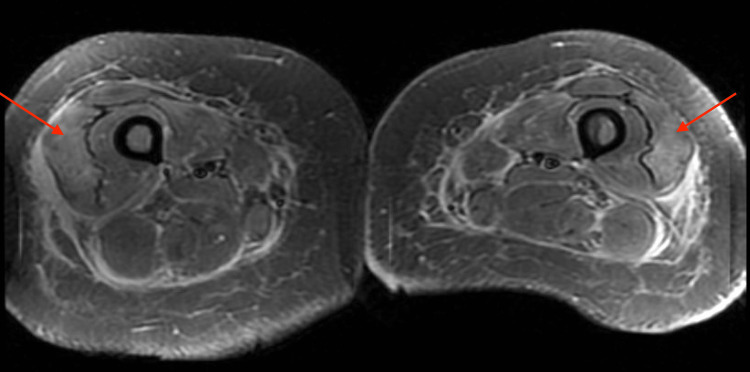
MRI of the lower extremities showing bilateral patchy hyperintense signal in the lateral compartment. Moderate myositis.

Flow cytometry was negative for malignancy. There was splenomegaly and diffused increase of the hepatic density found on the CT abdomen. An MRI of the lower extremities showed symmetric moderate myositis (Figure 3). Based on these findings, a presumptive diagnosis of dermatomyositis was made, and pulse dose steroids, intravenous immunoglobulin (IVIG), and cyclosporine were started. Despite these interventions, the patient developed a 20-fold increase in liver function tests and renal failure, requiring hemodialysis. Additionally, inflammatory markers drastically worsened, and the patient developed profound pancytopenia leading to the discontinuation of cyclosporine (Table 1). HLH workup yielded a >99% probability for HLH, and the patient was diagnosed with MAS-HLH. Chemokine (C-X-C motif) ligand 9 (CXCL9) and natural killer (NK) cell activity labs were drawn and sent to an outside reference lab, but they were canceled due to packaging errors.

For treatment, the patient was continued on pulse dose steroids, IVIG, and an interleukin-1 receptor antagonist (anakinra) was initiated. A skin biopsy found perivascular dermatitis and necrotic keratinocytes. Lymph node biopsy revealed necrotizing lymphadenopathy and an associated atypical T-cell lymphoproliferative process. Due to concerns for malignancy, T-cell gene arrangement was ordered, anakinra was discontinued, and etoposide was initiated. On this regimen, the patient exhibited significant clinical improvement both in symptomatology and laboratory indices. Her bone marrow biopsy confirmed HLH, and no evidence of neoplastic cells was found. With the results excluding malignancy, worsening pancytopenia, and improved clinical status, the patient only received five doses of etoposide. She was transitioned back to anakinra and continued to show improvement (Table 1). Eventually, the patient was transferred to the floor and discharged on a taper of steroids and anakinra. An outpatient muscle biopsy diagnosed her with immune-mediated necrotizing myopathy.

## Discussion

HLH is a rare condition that is classified as primary or secondary. Primary HLH, commonly seen in children, is due to genetic alterations. Secondary HLH is triggered by a secondary cause such as malignancy, infections, or rheumatological disorders [1]. Due to its rarity, the incidence of adult HLH is not well established. The diagnosis of HLH can be made based on the clinical presentation, specific genetic mutations, and/or fulfillment of the diagnostic criteria from the HLH-2004 trial [2]. The H score was developed to help with the diagnosis of HLH. Findings including fever, splenomegaly, peripheral blood cytopenia, hypertriglyceridemia/hypofibrinogenemia, hemophagocytosis on biopsy of hematopoietic tissue, low or absent NK cell activity, ferritin >500 ng/mL, elevated soluble CD25, and elevated CXCL9 are used to diagnose HLH [2,3]. Aside from verified HLH genetic mutations, the presence of five of the nine findings yields a diagnosis of HLH. In adult patients with a diagnosis of HLH, investigation of the cause is imperative. Currently, the leading cause of HLH is malignancy, followed by infection. One literature review of 162 adults with HLH found malignant in 60%, infection in 25%, and autoimmune in 3% [4]. In the setting of rheumatological disorders, MAS-HLH is synonymous with HLH. In the subset of rheumatological causes of MAS-HLH, systemic juvenile idiopathic arthritis, adult-onset Still disease, and SLE are the most common [1]. Reported cases of MAS-HLH secondary to inflammatory myopathies are exceedingly rare in the medical literature. To our knowledge, this is the first reported case of necrotizing myositis as a cause of MAS-HLH.

In a large retrospective case, a report of 424 patients with inflammatory myopathies, including dermatomyositis (DM), polymyositis (PM), and clinically amyopathic dermatomyositis (CADM), 18 patients (4.2%) fulfilled the 2004 diagnostic criteria for HLH. This study found a significantly higher short-term mortality rate in MAS-HLH patients when compared to their non-MAS-HLH counterparts (78% vs. 7%, respectively) [5]. It also established infection as a risk factor for the development of MAS-HLH, with 83% of MAS-HLH patients having an active infection at the time of diagnosis. Only 17% of the MAS-HLH diagnosed patients developed HLH exclusively from rheumatological disorders, emphasizing its rarity [5]. As is common in most cases of HLH, diagnosis and rapid initiation of treatment are the rate-limiting steps.

According to current treatment guidelines for unstable or deteriorating patients with HLH, steroids with or without IVIG should be initiated, with treatment then tailored based on etiology. It is recommended that patients with autoimmune-associated HLH be started on methylprednisolone and an interleukin-1 receptor antagonist, such as anakinra. If the response is insufficient or CNS involvement is present, etoposide is recommended [6]. A limited number of studies explore alternative therapies for inflammatory myopathy-associated MAS-HLH. A literature review identified 18 cases of dermatomyositis-associated MAS. In these cases, treatment with steroids alone was ineffective, but with the addition of rituximab, outcomes improved [7]. In one case report, the addition of cyclophosphamide to steroids and IVIG was found to be effective in decreasing ferritin, but the patient ultimately expired [8].

## Conclusions

Currently, the incidence of HLH in adults has not been well established, but mortality in reported cases is high and universally fatal if untreated. Literature regarding inflammatory myopathies-associated MAS-HLH shows a worse prognosis when compared to HLH due to other etiologies. Therefore, early recognition and treatment are paramount. In patients with inflammatory myopathies who continue to clinically worsen despite appropriate initial therapy, HLH should be high on the differential. If treatment with high-dose steroids and IVIG is ineffective, rapid escalation to an interleukin-1 receptor antagonist (anakinra) and chemotherapy (etoposide) should be considered.
